# Biomarker changes preceding symptom onset in genetic prion disease

**DOI:** 10.1101/2023.12.18.23300042

**Published:** 2023-12-18

**Authors:** Sonia M Vallabh, Meredith A Mortberg, Shona W. Allen, Ashley C Kupferschmid, Pia Kivisäkk, Bruno L Hammerschlag, Anna Bolling, Bianca A. Trombetta, Kelli Devitte-McKee, Abaigeal M. Ford, Lauren Sather, Griffin Duffy, Ashley Rivera, Jessica Gerber, Alison J McManus, Eric Vallabh Minikel, Steven E Arnold

**Affiliations:** 1. McCance Center for Brain Health and Department of Neurology, Massachusetts General Hospital, Boston, MA 02114; 2. Stanley Center for Psychiatric Research, Broad Institute of MIT and Harvard, Cambridge, MA 02142; 3. Department of Neurology, Harvard Medical School, Boston, MA 02115

## Abstract

**Importance.:**

Genetic prion disease is a universally fatal and rapidly progressive neurodegenerative disease for which genetically targeted therapies are currently under development. Preclinical proofs of concept indicate that treatment before symptoms will offer outsize benefit. Though early treatment paradigms will be informed by the longitudinal biomarker trajectory of mutation carriers, to date limited cases have been molecularly tracked from the presymptomatic phase through symptomatic onset.

**Objective.:**

To longitudinally characterize disease-relevant cerebrospinal fluid (CSF) and plasma biomarkers in individuals at risk for genetic prion disease up to disease conversion, alongside non-converters and healthy controls.

**Design, setting, and participants.:**

This single-center longitudinal cohort study has followed 41 *PRNP* mutation carriers and 21 controls for up to 6 years. Participants spanned a range of known pathogenic *PRNP* variants; all subjects were asymptomatic at first visit and returned roughly annually. Four at-risk individuals experienced prion disease onset during the study.

**Main outcomes and measures.:**

RT-QuIC prion seeding activity, prion protein (PrP), neurofilament light chain (NfL) total tau (t-tau), and beta synuclein were measured in CSF. Glial fibrillary acidic protein (GFAP) and NfL were measured in plasma.

**Results.:**

We observed RT-QuIC seeding activity in the CSF of three E200K carriers prior to symptom onset and death, while the CSF of one P102L carrier remained RT-QuIC negative through symptom conversion. The prodromal window of RT-QuIC positivity was one year long in an E200K individual homozygous (V/V) at PRNP codon 129 and was longer than two years in two codon 129 heterozygotes (M/V). Other neurodegenerative and neuroinflammatory markers gave less consistent signal prior to symptom onset, whether analyzed relative to age or individual baseline. CSF PrP was longitudinally stable (mean CV 10%) across all individuals over up to 6 years, including at RT-QuIC positive timepoints.

**Conclusion and relevance.:**

In this study, we demonstrate that at least for the E200K mutation, CSF prion seeding activity may represent the earliest detectable prodromal sign, and that its prognostic value may be modified by codon 129 genotype. Neuronal damage and neuroinflammation markers show limited sensitivity in the prodromal phase. CSF PrP levels remain stable even in the presence of RT-QuIC seeding activity.

## INTRODUCTION

Prion disease features striking biomarker signatures^1–4^, but limited data exist on pre-symptomatic changes^5–7^. Mirroring disease duration^8^, prodomal change in genetic prion disease appears brief, preceding symptoms by at most 1–4 years^6,7^. Prion “seeds” in CSF have been detected by real-time quaking induced conversion (RT-QuIC) in pre-symptomatic individuals^5,7^, but prognostic value remains unclear. Here, we report fluid biomarker trajectories associated with 4 disease onsets over 6 years in a longitudinal natural history of genetic prion disease mutation carriers.

## METHODS

### Study participants.

This previously described^5^ cohort study includes asymptomatic individuals with pathogenic *PRNP* mutations; individuals at risk for same; and controls (Table 1; Figure S1). Individuals with contraindication to lumbar puncture were excluded. Each visit included CSF and plasma collection, a medical history and physical, and a battery of cognitive, psychiatric, and motor tests and inventories. Individuals were invited to complete a baseline visit, a short-term repeat 2–4 months later (pre-2020), and approximately yearly visits thereafter. Data presented here were collected July 2017 to February 2023 and include data previously reported^5,9^. All participants were cognitively normal and provided written informed consent. This study was approved by the Mass General Brigham Institutional Review Board (2017P000214). Assay validation utilized samples from MIND Tissue Bank (2015P000221).

### Biomarker assays.

Biomarker assays utilized were: RT-QuIC (IQ-CSF protocol)^10^, PrP ELISA^9^, Simoa (Quanterix) GFAP, and Ella (Bio-Techne) NfL, T-tau (Figure S3), and β-syn (Figure S4), see Supplementary Methods.

### Statistical analysis.

Biomarker relationships with age and mutation status were assessed by log-linear regression; curve fits shown in figures are the separate best fits for mutation carriers and for controls, while P values are for the effect of carrier status in a combined model: lm(log(value) ~ age + carrier). Our study does not disclose biomarker values or *PRNP* mutation status to participants, yet a combination of age and the number and spacing of visits completed could uniquely identify some individuals, presenting a selfidentification risk. To mitigate this risk, for controls and non-converting carriers in data visualizations, ages were obfuscated by addition of a normally distributed random variable with mean of 0 and standard deviation of ±3 years, and visit spacing intervals were obfuscated by multiplication by a normally distributed random variable with mean 1 and standard deviation ±25%, capped at a maximum increase of +25% to avoid visually exaggerating the study’s duration. True ages and true visit intervals for all participants are used in all descriptive statistics and statistical models and true ages and true visit intervals are shown in plots for the individuals who converted to active disease. For details of RT-QuIC analysis see Supplementary Methods. P values <0.05 were considered nominally significant. Analyses were conducted in R 4.2.0. Source code, summary statistics for all participants, and individual biomarker values for converting participants are available at https://github.com/ericminikel/mgh_prnp_freeze2

## RESULTS

**Figure 1.**
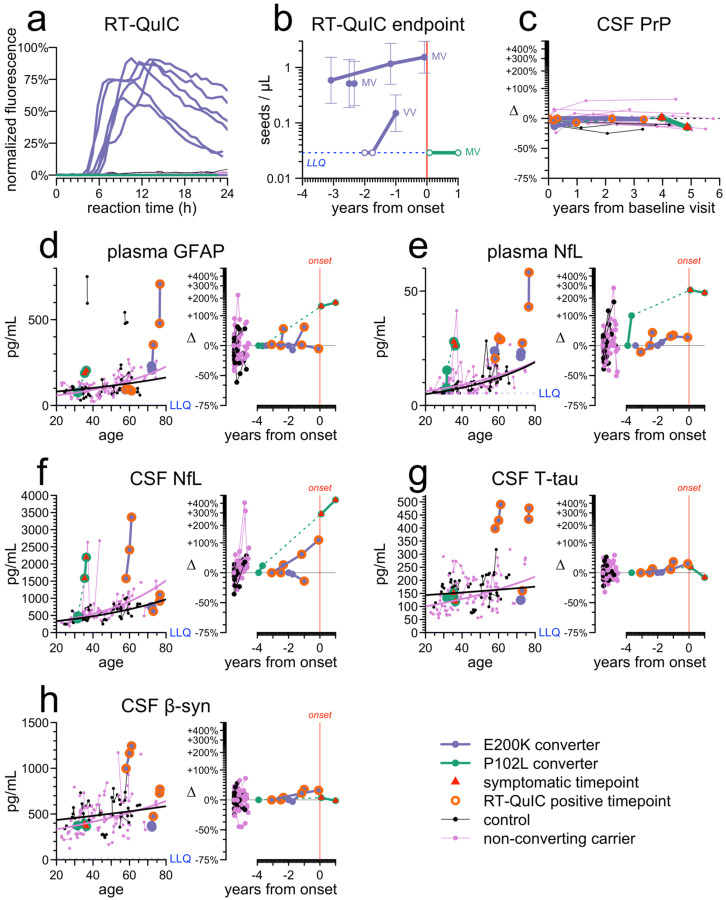
Fluid biomarker changes in the cohort. **A)** RT-QuIC kinetic curves, showing 6 positive CSF samples (each with 4/4 replicates positive) out of 149 tested (98 from carriers, 51 from controls). **B)** RT-QuIC endpoint titration of CSF, with codon 129 genotypes of converters indicated. **C)** CSF PrP concentrations represented as changes (Δ) relative to individual baseline, shown for the 4 converters plus all individuals (N=11) with at least 3 years between first and last available CSF sample. **D-H)** Biomarkers plasma GFAP (**D**), plasma NfL (**E**), CSF NfL (**F**), CSF T-tau (**G**), and CSF β-syn (**H**) are represented by two views each. Left: individual age vs. absolute concentration in pg/mL, with sequential samples from the same individual connected by thin lines, while thicker lines represent the separate log-linear best fit curves for controls and for non-converting carriers. Right: years from disease onset vs. change (Δ) relative to individual baseline in converters, with the same for controls and for non-converting carriers shown on a separate x-axis. Dashed lines connect timepoints before and after symptom onset.

Of 41 carriers (Table 1), four converted to active disease (N=3 E200K, N=1 P102L). 6 RT-QuIC positives (Figure 1A) belonged to 3 E200K individuals who converted and died of prion disease. 2 *PRNP* codon 129 heterozygotes (M/V) were RT-QuIC positive at first sample (2.5 and 3.1 years before onset); prion titer in CSF did not appreciably rise thereafter (Figure 1B). One homozygote (V/V) became RT-QuIC positive on study and became symptomatic 1 year later.

Plasma GFAP, a marker of reactive astrogliosis, was high relative to age in 2/4 converters, but change from individual baseline was unremarkable compared to controls and non-converters (Figure 1D). Plasma NfL appeared high and increased in all 4 converters, but not outside the range of non-converters and controls (Figure 1E). CSF NfL, CSF t-tau, and CSF beta-synuclein were each elevated in 2/4 converters and normal in 2/4 (Figure 1F–H); different converting individuals were high for different markers.

## DISCUSSION

Here we describe fluid biomarker profiles in a longitudinal cohort of genetic prion disease mutation carriers, including 4 individuals who converted to active disease. As before^5–7^, at any given time, cross-sectionally, most carriers of genetic prion disease mutations do not have any detectable molecular sign of disease. Our data support the hypothesis that CSF prion seeding activity as assayed by RT-QuIC may represent the first detectable change in E200K carriers. However, we did not detect seeding activity in the CSF of a P102L converter, consistent with RT-QuIC’s lower sensitivity for most non-E200K genetic subtypes^1,11^. Though our sample is small, our data suggest that PRNP codon 129 genotype may modify the duration of CSF RT-QuIC positivity before onset in E200K individuals; longer prodromal positivity in M/V heterozygotes would mirror their longer disease duration after onset^12^.

Soluble PrP in CSF is reduced in symptomatic prion disease patients, presumably as a result of a disease sink process^13–16^, and yet pharmacologic lowering of CSF PrP may be important as a drug activity biomarker for trials of PrP-lowering drugs, and has been proposed as a surrogate endpoint in prevention trials^17^. Our data suggest that CSF PrP does not begin to decline prior to symptom onset, even in the presence of RT-QuIC positivity, suggesting its use in asymptomatic individuals will not be confounded.

Neuronal damage and neuroinflammation markers rise with age and may vary between individuals. Neither when normalized to age nor to individual baseline did any of these markers consistently provide distinctive signal in all 4 of our converting individuals relative to non-converters and controls. Thus, while these markers may be useful as an adjunct, none is likely to provide the prognostic specificity of RT-QuIC. RT-QuIC, meanwhile, may offer just 1 year of advance signal in some E200K cases, and currently faces limited sensitivity to other subtypes. Assay improvement, biomarker discovery, and continued sample accrual will be vital to identifying additional prognostic markers, particularly for non-E200K subtypes. At any given time, most carriers appear non-prodromal, thus, in this rare disease, prodromal individuals are unlikely to be identified in sufficient numbers to power clinical trials. Instead, primary prevention trials with inclusion based on genotype and CSF PrP as primary endpoint may be necessary^17^, and would honor the outsize benefit of early treatment observed in animal models^18^. Treatment of prodromal individuals could feature as a supportive arm and/or randomization off-ramp for carriers who develop a prodromal signature during a trial.

### Limitations.

Four symptom onsets is a small absolute number from which to draw conclusions. Reflecting study enrollment and overall mutation prevalence, our observed onsets are skewed towards E200K. Some annual visits were missed due to COVID-19. We did not collect emerging sample types such as nasal brushings^19^, urine^20^, or tears^21^. Additional pre-symptomatic natural history work across multiple sites^7,22,23^ will be required to build confidence in our observations.

## CONCLUSIONS

In E200K carriers, RT-QuIC seeding activity in CSF can precede symptom onset by 1–3 years, perhaps depending on *PRNP* codon 129 genotype. CSF and plasma markers of neurodegeneration and neuroinflammation do not unambiguously identify imminent converters. CSF PrP levels are longitudinally stable over time in all participants even following RT-QuIC positivity.

## Supplementary Material

Supplement 1

2

## Figures and Tables

**Table 1.** Demographic characteristics of the cohort. “Age” represents age last seen, follow-up is years from first visit to last visit, and both are represented by mean ± SD.

mutation status	N	sex	age (y)	follow-up (y)	total visits	CSF samples	plasma samples	mutations
carrier	41	13M / 28F	47.5±14.0	2.0±1.9	126	104	109	6 P102L 7 D178N 22 E200K 6 other
control	21	6M / 15F	46.1±13.3	1.4±1.5	57	51	51	21 none

CSF total PrP levels varied between individuals and were lower in mutation carriers (Figure S2) but were longitudinally stable in each individual out to 6 years (mean CV 10%) (Figure 1C), including samples taken after RT-QuIC positivity.

## KEY POINTS

### Question.

What biofluid-based molecular changes precede symptom onset in genetic prion disease? For any observed changes, how consistently and by how long do they precede onset?

### Findings.

In this longitudinal study, presymptomatic CSF RT-QuIC prion seeding activity was observed in three E200K carriers who subsequently developed and died of prion disease, with changes in prodromal timing noted based on *PRNP* codon 129 genotype. CSF PrP levels were longitudinally stable in all study participants up to 6 years, regardless of mutation status or presence of RT-QuIC seeding activity.

### Meaning.

These findings suggest that CSF RT-QuIC may offer a brief, genotype-dependent prodromal signal prior to symptom onset in carriers of the E200K mutation. They further indicate that across *PRNP* mutations, CSF PrP levels are sufficiently stable to serve as a drug activity biomarker for PrP-lowering therapeutics and will not be confounded by disease-related molecular changes that precede symptom onset.
