# Supplementary material for: Biomarker changes preceding symptom onset in genetic prion disease
Source: medRxiv. 2023 Dec 18:2023.12.18.23300042. Preprint. [Version 1] doi: 10.1101/2023.12.18.23300042 (PMC10775317; doi:10.1101/2023.12.18.23300042)
Supplement: 2 [file NIHPP2023.12.18.23300042V1-supplement-1.pdf]

medRxiv preprint doi: https://doi.org/10.1101/2023.12.18.23300042; this version posted December 18, 2023. The copyright holder for this preprint (which was not certified by peer review) is the author/funder, who has granted medRxiv a license to display the preprint in perpetuity.

It is made available under a CC-BY 4.0 International license .

SUPPLEMENT

Biomarker changes preceding symptom onset in genetic prion disease

Sonia M Vallabh[1,2,3,†], Meredith A Mortberg[1,2], Shona W. Allen[1], Ashley C Kupferschmid[1], Pia K Webb[1], Bruno L Hammerschlag[1], Anna Bolling[1], Bianca A. Trombetta[1], Kelli Devitte-McKee[1], Abigeal M. Ford[1], Griffin Duffy[1], Ashley Rivera[1], Jessica Gerber[1], Alison J McManus[1], Eric Vallabh Minikel[1,2,3], Steven E Arnold[1,3,†]

Table of Contents

Supplementary Methods 12

Supplementary Figures 14

- **Figure S1. Flow chart of participant recruitment.** 14
- **Figure S2. CSF PrP concentration by PRNP mutation.** 15
- **Figure S3. Quality control analyses on the Ella T-tau assay.** 16
- **Figure S4. Quality control analyses on the beta-synuclein assay.** 17

Supplementary Tables 18

- **Table S1. All biomarker values from all visits by individuals who developed active disease.** 18
- **Table S2. Means, standard deviations, and ranges of biomarker values from all visits by participants who did not develop active disease, by mutation status.** 19
- **Table S3. Mean CSF PrP concentration (ng/mL) by mutation.** 19
- **Table S4. Long-term test-retest reliability of CSF PrP.** 20
- **Table S5. Descriptive statistics and log-linear model fits on CSF and plasma biomarkers.** 20

STROBE checklist 21

medRxiv preprint doi: https://doi.org/10.1101/2023.12.18.23300042; this version posted December 18, 2023. The copyright holder for this preprint (which was not certified by peer review) is the author/funder, who has granted medRxiv a license to display the preprint in perpetuity.

It is made available under a CC-BY 4.0 International license .

Supplementary Methods

Genotyping. Whole blood was frozen hemolyzed and genomic DNA was extracted. All samples were genotyped by two orthogonal methods. DNA was submitted for targeted capture using a custom set of probes (Twist Biosciences) directed against ~150 kb of genomic sequence in and surrounding *PRNP*, then enriched DNA was subjected to deep short-read sequencing (Illumina) at the Broad Institute's Genomics Platform. Data were aligned to the hg38 reference genome and processed using Dragen 3.7.8 to yield multi-sample VCF files. In parallel, DNA also underwent a previously described[24] protocol implemented by Genewiz, combining Sanger sequencing to detect SNPs and short indels with gel sizing to detect octapeptide repeat insertions (OPRI). Briefly, the primers utilized are: Int5: 5′-TgCATgTTTTCACgATAgTAACgg-3′, DG2: 5′-gCAgTCATTATggCgAACCTTggCTg-3′, and 3′Sal: 5′-gTACTgAggATCCTCCTCATCCCACTATCAggAAgA-3′. The prodcut of the DG2/3′Sal reaction is subjected to Sanger sequencing; the product of the DG2/Int5 reaction is run on a 2% agarose gel (the wild-type product is 464 bp). Genotypes obtained by the two different methods were in agreement for all samples. Determination of haplotypes was accomplished by molecular phasing of codon 129 to pathogenic variants by paired-end Illumina sequencing reads using a custom Python 3 script run on Terra (Terra.bio); source code is available in the study's online GitHub repository. Our study includes individuals who are at risk for inheriting a *PRNP* mutation but have not undergone predictive testing; genotypes were determined for research purposes only and were not disclosed to participants.

Sample processing. Blood was collected in purple top K+ EDTA tubes, inverted gently, and centrifuged at 1,500 *g* for 10 minutes to retrieve plasma, aliquoted, and frozen at -80°C. 20 mL of CSF was collected via gentle aspiration lumbar puncture using a 24G atraumatic Sprotte needle into 4x 5 mL syringes. Because PrP in CSF is highly sensitive to polypropylene adsorption, we followed the protocol described in Vallabh 2019 Figure S8, where 2 of the 4 collected 5 mL aliquots were ejected into tubes pre-loaded with the zwitterionic detergent CHAPS (3% wt/vol stock solution at 1% volume to yield a final 0.03% CHAPS concentration). All CSF were centrifuged at 2,000 *g* for 10 minutes to remove cells, and then aliquoted and frozen at -80°C. In instances where the LP yielded only a limited volume of CSF, CHAPS aliquots were prioritized. CHAPS aliquots were used for PrP and NfL quantification. Neat aliquots were used for RT-QuIC. For T-tau and beta-synuclein, neat aliquots were used where available, while CHAPS aliquots were used when these were the only available samples; an assessment of the effect of 0.03% CHAPS on these assays is provided in Figures S3-S4. To minimize bias, technicians processing samples and performing biomarker assays were blinded to genotype.

RT-QuIC. Real-time quaking-induced conversion (RT-QuIC) was performed according to the protocol of Orru et al 2015, widely referred to as the IQ-CSF protocol[10]. The substrate was recombinant N-terminally truncated Syrian hamster PrP (SHaPrP90-230) expressed in *E. coli* and produced in-house according to a published protocol[25,26] and filtered by centrifugation at 3,214 *g* through a 100 kDa filter (PALL OD100C33). Final concentration in the reaction was 300 mM NaCl (Broad Institute SQM), 10 mM sodium phosphate pH 7.4 (Molecular Toxicology; Thermo C790B91), 1 mM EDTA (Broad Institute SQM), 10 µM thioflavin T (Sigma T3516-5G), 0.002% sodium dodecyl sulfate (SDS) (Invitrogen 15553-035), and 0.1 mg/mL recombinant PrP, all diluted into distilled water (InvitroGen UltraPure 10977-015). 80 µL of a 1.25x concentrated master mix was loaded into each well of a 96-well plate (Nunc; Thermo 265301) and then 20 µL of CSF was added. Plates were sealed with adhesive film (VWR 37000-548). The assay was run at 55°C for 24 hours on a BMG FLUOStar OPTIMA platereader with alternating cycles of 1 minute rest and 1 minute 800 rpm shaking, with

medRxiv preprint doi: https://doi.org/10.1101/2023.12.18.23300042; this version posted December 18, 2023. The copyright holder for this preprint (which was not certified by peer review) is the author/funder, who has granted medRxiv a license to display the preprint in perpetuity.

It is made available under a CC-BY 4.0 International license .

thioflavin T fluorescence measurements obtained via bottom read at 45-minute intervals with 450 nm excitation and 480 nm emission. Fluorescence kinetic curves were normalized so that 0% represents the baseline fluorescence value at first reading and 100% represents the instrument's maximum value of 65,000 fluorescence units. We committed to the pre-specified criteria of Orru et al[10]: a CSF sample was called positive if at least 50% of technical replicates (e.g. 2/4) yielded at least 10% normalized signal within 24 hours. In practice, when screening undiluted CSF, all our positive samples were positive in 4/4 replicates while all negatives were positive in 0/4 replicates. For endpoint titration, 3-fold serial dilutions were run by adding 20, 6.7, 2.2, or 0.7 µL of CSF and then 0, 13.3, 17.8, or 19.3 µL of distilled water (InvitroGen UltraPure 10977-015). Titers were determined by Spearman-Karber analysis[27]; the source code is available in this study's online GitHub repository.

PrP ELISA. PrP enzyme-linked immunosorbent assay (ELISA) was performed according to an in-house protocol previously published and described in detail[9]. The assay uses antibodies EP1802Y (Abcam ab52604) for capture and 8H4 (Abcam ab61409), biotinylated in-house, for detection. The standard curve is recombinant full-length mouse PrP (MoPrP23-231) produced in house, plated at concentrations from 0.05 ng/mL to 5 ng/mL. CSF was run at a dilution factor of 80, at which the lower limit of quantification (LLOQ) is 4 ng/mL. For longitudinal analysis (Figure 1C, Table S4), each individual was normalized to their own baseline. For comparison across mutations (Figure S2, Table S3), all individuals were normalized to the mean value in mutation-negative subjects, which was 70.6 ng/mL.

GFAP. Plasma GFAP was quantified using Simoa (Quanterix) according to manufacturer instructions at a dilution factor of 4, yielding an LLOQ of 2.744 pg/mL. Samples were run in technical duplicate with a mean CV of 6.0%.

NfL. Plasma and CSF NfL were quantified using Ella by ProteinSimple (Bio-Techne) at a dilution factor of 2 yielding an LLOQ of 5.4 pg/mL. CSF aliquots containing CHAPS were used. For all Ella assays, samples were plated onto cartridges in singlicate; each sample is then run in technical triplicate with three glass nanoreactors (GNRs).

T-tau. CSF T-Tau was analyzed both by ELISA (Fujirebio) and by Ella (Bio-Techne). For Ella, samples were run at a dilution factor of 2 (except for N=6 samples run at a dilution factor of 3 due to limited volume), with an LLOQ of 1.68 pg/mL. For ELISA, samples were run at a dilution factor of 4, with an LLOQ of 39.5 pg/mL. Ella results are reported in Figure 1G, while a comparison of the two assays is given in Figure S3.

Beta-synuclein. CSF beta-synuclein was analyzed by Ella (Bio-Techne) at a dilution factor of 2 for CSF (LLOQ: 15.9 pg/mL) and either 4 or 8 for plasma depending on available sample volume (LLOQ: 31.8 pg/mL or 63.7 pg/mL respectively). As shown in Figure S4, all plasma samples from study participants were at LLOQ.

medRxiv preprint doi: https://doi.org/10.1101/2023.12.18.23300042; this version posted December 18, 2023. The copyright holder for this preprint (which was not certified by peer review) is the author/funder, who has granted medRxiv a license to display the preprint in perpetuity.

It is made available under a CC-BY 4.0 International license .

Supplementary Figures

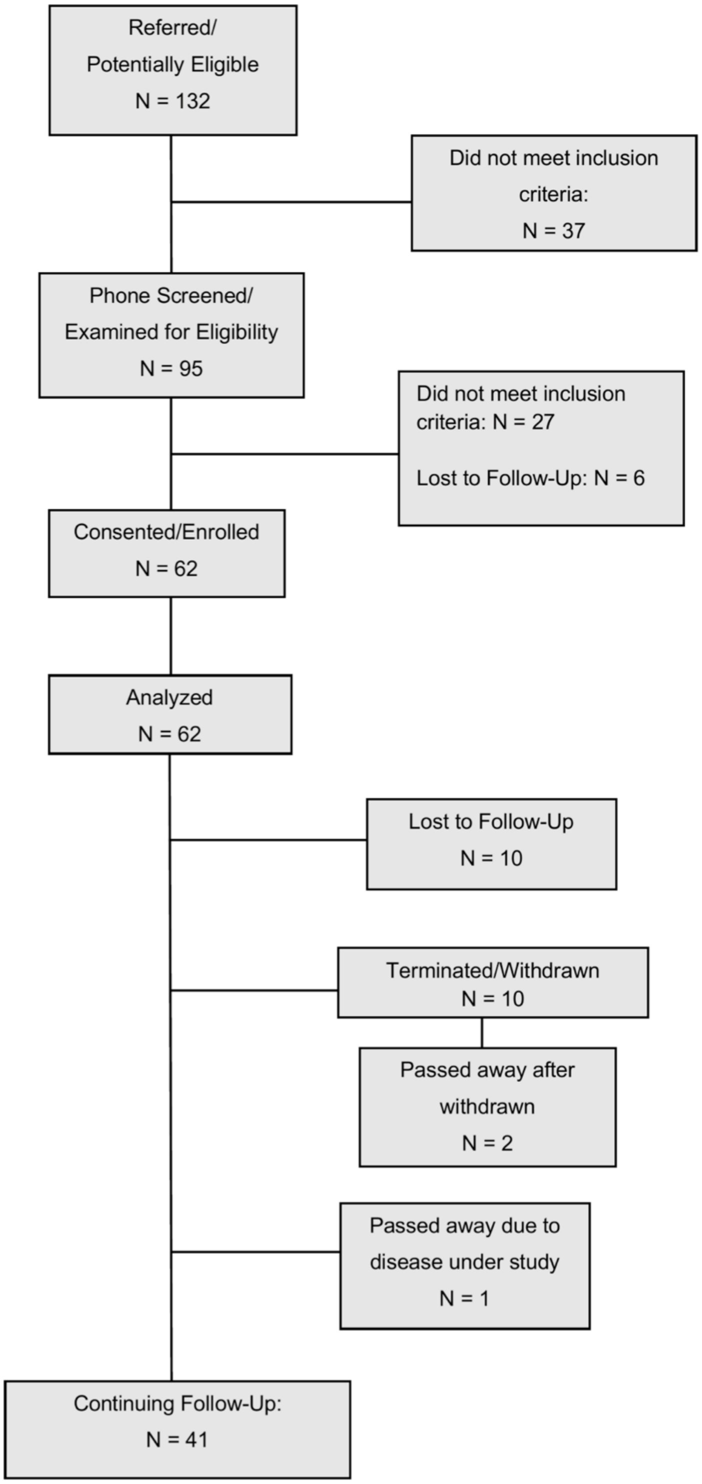

Figure S1. Flow chart of participant recruitment.

At launch in July 2017, the study was open to known mutation carriers, those at risk, and known controls. From November 2021 new enrollment restricted to only known carriers, but already-enrolled individuals were invited to continue to participate regardless.

medRxiv preprint doi: https://doi.org/10.1101/2023.12.18.23300042; this version posted December 18, 2023. The copyright holder for this preprint (which was not certified by peer review) is the author/funder, who has granted medRxiv a license to display the preprint in perpetuity.

It is made available under a CC-BY 4.0 International license .

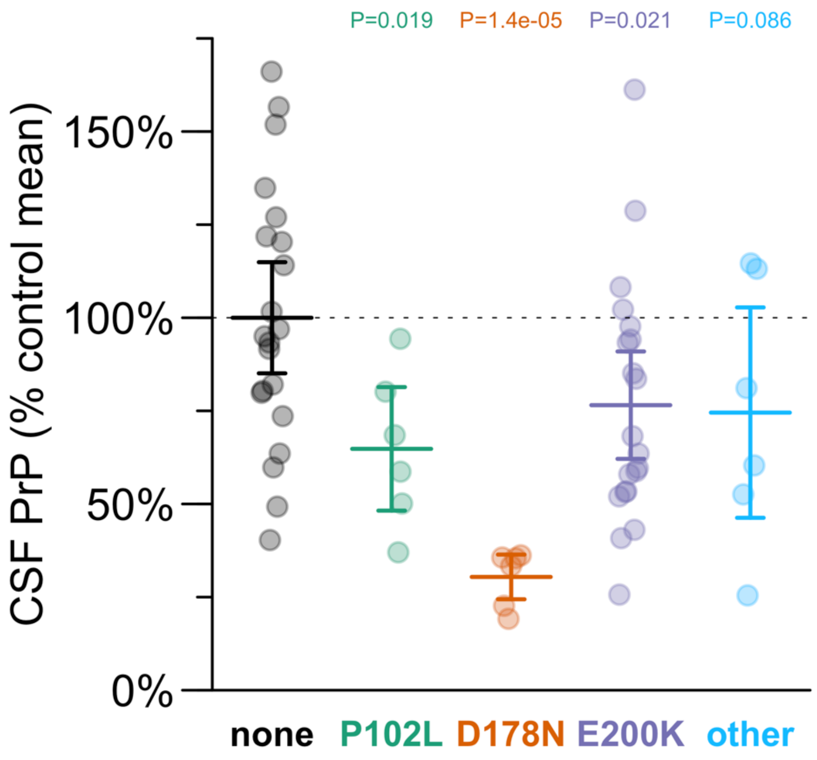

Figure S2. CSF PrP concentration by PRNP mutation.

Each point represents the mean of all available CSF samples for one study participant. Data are normalized to the mean of the mutation-negative controls ("none"). P values are for differences from the control group in a linear model (lm in R, equivalent to Type I ANOVA).

medRxiv preprint doi: https://doi.org/10.1101/2023.12.18.23300042; this version posted December 18, 2023. The copyright holder for this preprint (which was not certified by peer review) is the author/funder, who has granted medRxiv a license to display the preprint in perpetuity.
It is made available under a CC-BY 4.0 International license .

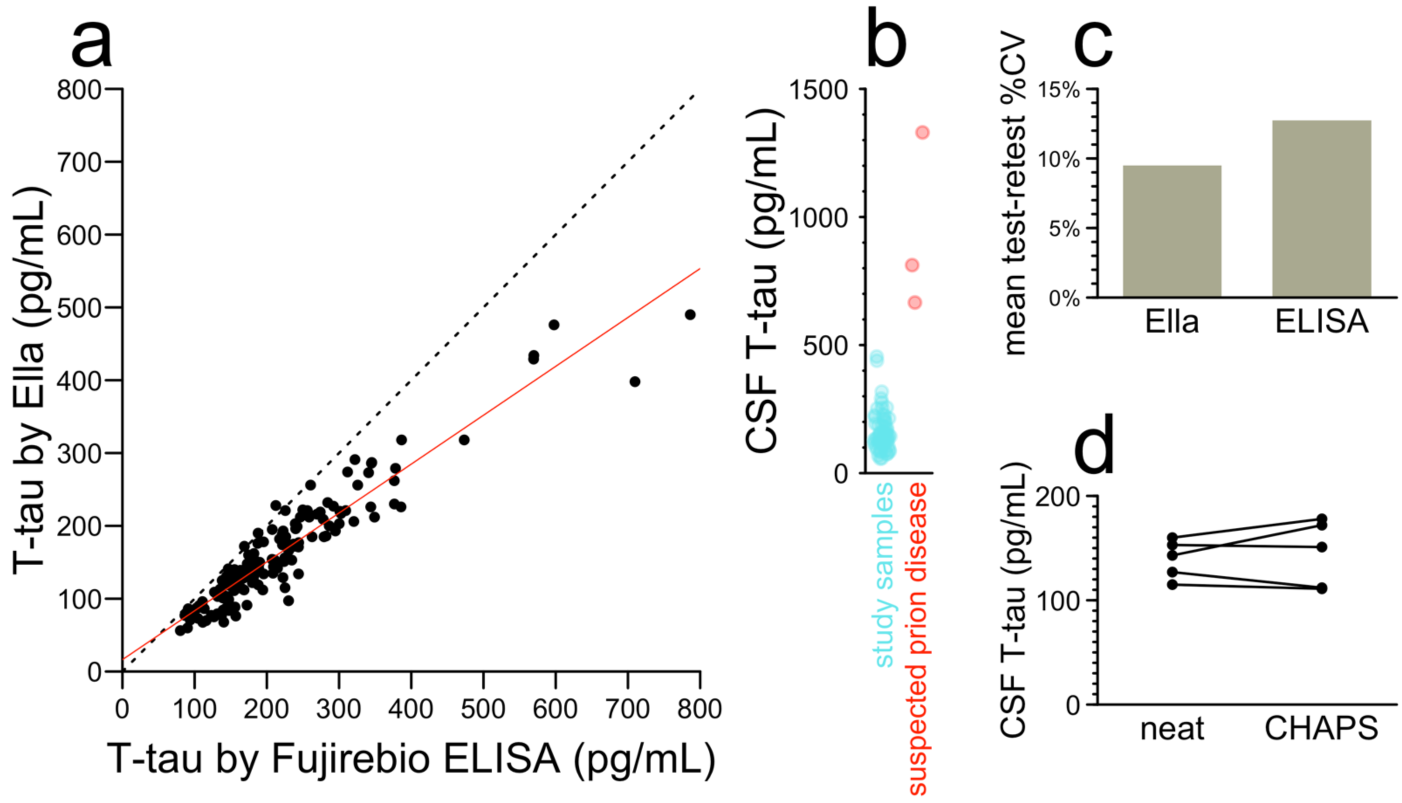

Figure S3. Quality control analyses on the Ella T-tau assay.

A) *Comparison of CSF T-tau concentrations in pg/mL for N=151 CSF samples determined by Fujirebio ELISA (x axis) versus Ella (y axis). The red line shows the best fit linear regression which is ella = 16.3 pg/mL + 67% × elisa. The Pearson's correlation is r = 0.94, P = 5.3e-70.* ***B)*** *Comparison of mean CSF T-tau values per individual by Ella in study participants vs. 3 symptomatic patients with suspected prion disease.* ***C)*** *Mean test-retest CV for longitudinal LPs from the same individual: Ella 9.5%, ELISA 12.7%.* ***D)*** *Paired analysis of N=5 CSF samples analyzed by T-tau Ella both with and without the addition of 0.03% CHAPS. Mean value with CHAPS is 3% higher, P = 0.55 by paired T test.*

medRxiv preprint doi: https://doi.org/10.1101/2023.12.18.23300042; this version posted December 18, 2023. The copyright holder for this preprint (which was not certified by peer review) is the author/funder, who has granted medRxiv a license to display the preprint in perpetuity.

It is made available under a CC-BY 4.0 International license .

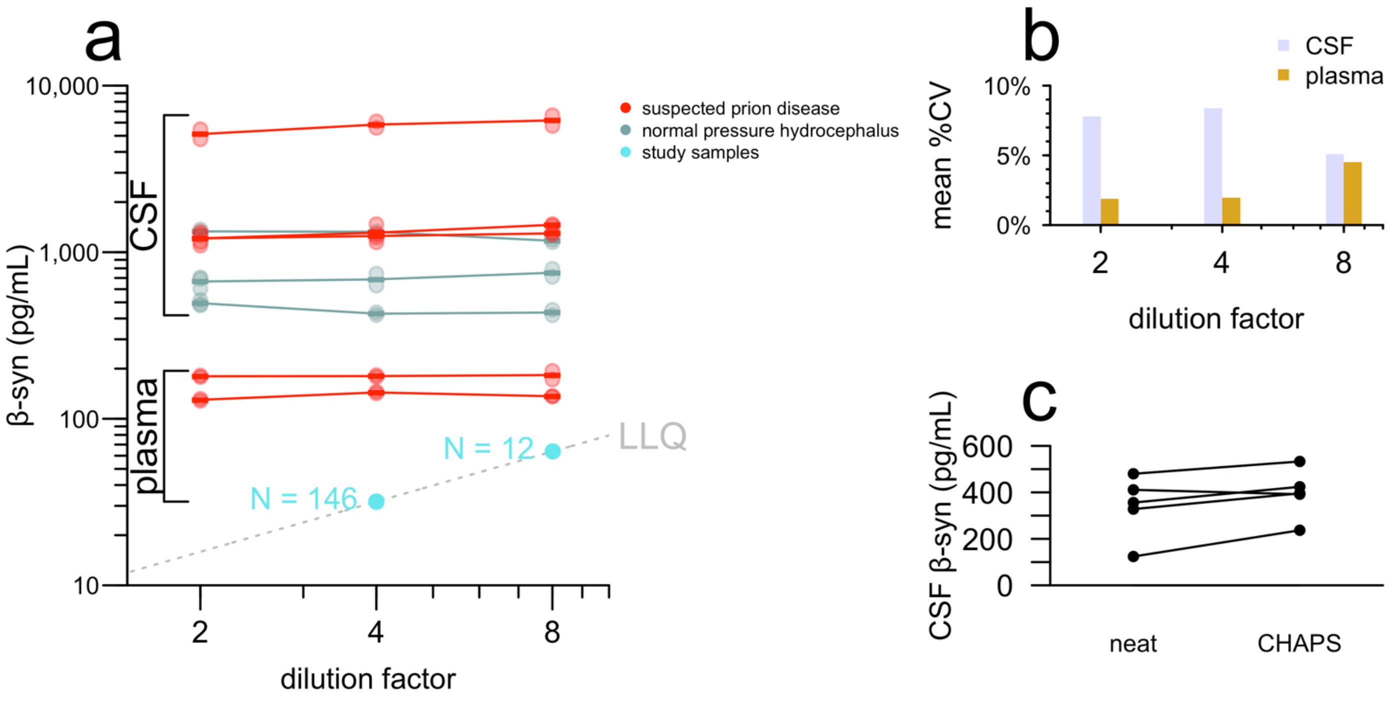

Figure S4. Quality control analyses on the Ella beta-synuclein assay.

A) *Parallelism (also called dilution linearity) tested on 6 CSF samples (3 suspected prion disease and 3 normal pressure hydrocephalus) and 2 plasma samples (2 suspected prion disease). Suspected prion disease patients are symptomatic individuals seen clinically at Massachusetts General Hospital outside of our study. All plasma samples from participants in our study were at the lower limit of quantification (LLQ).* ***B)*** *Mean coefficient of variation among 2 technical replicates per sample for the samples shown in (A). Note that this refers to plating the same sample twice, in separate wells, on the Ella cartridge; the measurement in each well is in turn the average of 3 replicate measurements.* ***C)*** *Comparison of 5 CSF samples from study participants analyzed both with and without the addition of 0.03% CHAPS, a detergent shown to reduce loss of PrP to plastic. Mean 27% higher reading in CHAPS samples, P = 0.057, paired T-test.*

medRxiv preprint doi: https://doi.org/10.1101/2023.12.18.23300042; this version posted December 18, 2023. The copyright holder for this preprint (which was not certified by peer review) is the author/funder, who has granted medRxiv a license to display the preprint in perpetuity.

It is made available under a CC-BY 4.0 International license .

Supplementary Tables

Supplementary tables are also available in the attached Excel spreadsheet and as tab-separated text files in the study's online GitHub repository.

Table S1. All biomarker values from all visits by individuals who developed active disease.

Genotype shows the pathogenic variant and codon 129. The 129MV individuals in this table are all cis-129M, trans-129V. Months from onset is negative for visits prior to symptom onset and positive for visits after symptom onset. Blank cells indicate assays not done because samples not collected (unsuccessful LP or virtual visit) or due to limited sample volume. MoCA[28] and MRC Scale[29] have been described elsewhere.

Individual	Genotype	Age of onset bin	Visit number	Months from onset	CSF RT-QuIC	CSF RT-QuIC replicates	CSF T-tau (pg/mL)	CSF NfL (pg/mL)	CSF β-syn (pg/mL)	CSF PrP (ng/mL)	Plasma NfL (pg/mL)	Plasma GFAP (pg/mL)	MoCA score	MRC Scale
A	E200K MV	75-79	1	-30	+	4/4	434	918	728	73.0	43.1	478.0	27	20
			2	-28	+	4/4	476	1103	771	69.8	58.2	707.7	25	20
B	P102L MV	35-39	1	-47				406	373	37.5	7.7	75.4	26	20
			2	-44			133	478		33.9	15.4	74.8	27	20
			3	1	-	0/4	152	1584	392	38.1	27.9	187.9	27	20
			4	12	-	0/4	119	2194	366	30.6	26.1	204.0	28	19
C	E200K VV	65-69	1	-24	-	0/4	124	757	372	32.8	21.4	229.6	29	20
			2	-21	-	0/4	125	732	358	27.6	23.1	207.2	28	20
			3	-12	+	4/4	160	623	474	29.9	27.3	353.7	24	20
D	E200K MV	60-64	1	-41							23.8	91.8	26	20
			2	-37	+	4/4	398	1575	997	66.4	20.5	94.1	26	20
			3	-16									26	20
			4	-14	+	4/4	429	2413	1164	66.2	29.5	93.1	27	20
			5	-1	+	4/4	490	3365	1244	64.8	28.8	85.3	29	20

medRxiv preprint doi: https://doi.org/10.1101/2023.12.18.23300042; this version posted December 18, 2023. The copyright holder for this preprint (which was not certified by peer review) is the author/funder, who has granted medRxiv a license to display the preprint in perpetuity.

It is made available under a CC-BY 4.0 International license .

Table S2. Means, standard deviations, and ranges of biomarker values from all visits by participants who did not develop active disease, by mutation status.

These summary statistics exclude all visits from the 4 participants who converted to active disease. In each cell, the top row shows mean±SD, while the bottom row shows range (min-max). CSF RT-QuIC positive shows the number of CSF samples that yielded an overall positive call. Each RT-QuIC reaction was run in quadruplicate; in this study, every positive sample was positive in all 4/4 replicates, while every negative sample was positive in 0/4 replicates.

Group	N visits	CSF RT-QuIC positive	CSF T-tau (pg/mL)	CSF NfL (pg/mL)	CSF β-syn (pg/mL)	CSF PrP (ng/mL)	Plasma NfL (pg/mL)	Plasma GFAP (pg/mL)	MoCA	MRC
Mutation-negative control	57	0/51	164±49<br>(75-318)	573±208<br>(242-1018)	516±144<br>(243-952)	71±24<br>(27-120)	9.7±4.6<br>(5.4-28.2)	145.9±150.1<br>(30.6-751.1)	28±3<br>(14-30)	20±0<br>(17-20)
Non-converting mutation carrier	112	0/88	149±65<br>(56-318)	685±404<br>(192-2677)	472±201<br>(124-1171)	45±24<br>(10-126)	11.1±6.6<br>(5.4-41.4)	112.8±53.3<br>(22.3-267.5)	28±2<br>(22-30)	20±0<br>(19-20)

Table S3. Mean CSF PrP concentration (ng/mL) by mutation.

These are the numeric values for the data shown in Figure S2. Results were grouped first by individual to determine mean CSF PrP concentration across longitudinal CSF samples, then grouped by mutation to determine mean and SD across individuals. N is the number of individuals in each group.

Mutation	N	Mean	SD	Normalized mean	Linear regression P value
none	21	69.9	24.4	100.0%	—
P102L	6	45.3	14.5	64.8%	1.92e-02
D178N	6	21.3	5.2	30.4%	1.42e-05
E200K	20	53.5	23.0	76.5%	2.06e-02
other	6	52.1	24.7	74.5%	8.64e-02

medRxiv preprint doi: https://doi.org/10.1101/2023.12.18.23300042; this version posted December 18, 2023. The copyright holder for this preprint (which was not certified by peer review) is the author/funder, who has granted medRxiv a license to display the preprint in perpetuity.

It is made available under a CC-BY 4.0 International license .

Table S4. Long-term test-retest reliability of CSF PrP.

Summary of data from Figure 1C. Test-retest mean CV summarized for all non-converting study participants with ≥3 years of longitudinal CSF data.

Group	N individuals	N samples total	Mean CV
non-converting carrier	12	52	9.71%
control	3	12	10.20%

Table S5. Descriptive statistics and log-linear model fits on CSF and plasma biomarkers.

						Overall model lm(log(value) ~ age + carrier, data = all)				
Biomarker	N individuals	N samples	Carrier samples	Control samples	Test-retest mean CV without converters	Y intercept	Annual increase	Annual increase P value	Carrier difference	Carrier difference P value
CSF NfL	60	155	104	51	16.3%	183.69	2.3%	8.1e-20	0.0028	0.0028
CSF T-tau	60	151	100	51	9.6%	101.29	0.9%	2.8e-04	0.0734	0.0734
CSF β-syn	60	150	99	51	10.8%	329.05	0.9%	3.8e-04	0.0819	0.0819
plasma NfL	62	160	109	51	19.6%	3.51	2.0%	9.3e-14	0.0558	0.0558
plasma GFAP	61	158	107	51	18.4%	45.78	1.9%	6.3e-08	0.6014	0.6014

	Model of non-converting carriers only lm(log(value) ~ age, data = NCcarriers)			Model of controls only lm(log(value) ~ age, data = controls)		
Biomarker	Y intercept	Annual increase	Annual increase P value	Y intercept	Annual increase	Annual increase P value
CSF NfL	193.9	2.6%	2.7e-15	233.9	1.8%	5.4e-06
CSF T-tau	77.5	1.3%	2.8e-04	133.9	0.3%	0.35
CSF β-syn	267.5	1.1%	0.0014	393.6	0.5%	0.13
plasma NfL	4.2	1.9%	1.9e-07	3.2	2.3%	1.0e-09
plasma GFAP	37.1	2.3%	6.2e-10	64.1	1.2%	0.11

medRxiv preprint doi: https://doi.org/10.1101/2023.12.18.23300042; this version posted December 18, 2023. The copyright holder for this preprint (which was not certified by peer review) is the author/funder, who has granted medRxiv a license to display the preprint in perpetuity.

It is made available under a CC-BY 4.0 International license .

STROBE checklist

STROBE Statement—Checklist of items that should be included in reports of ***cohort studies***

	Item No	Recommendation	Page No
**Title and abstract**	1	(*a*) Indicate the study's design with a commonly used term in the title or the abstract	1
		(*b*) Provide in the abstract an informative and balanced summary of what was done and what was found	1
**Introduction**			
Background/rationale	2	Explain the scientific background and rationale for the investigation being reported	1
Objectives	3	State specific objectives, including any prespecified hypotheses	1
**Methods**			
Study design	4	Present key elements of study design early in the paper	2
Setting	5	Describe the setting, locations, and relevant dates, including periods of recruitment, exposure, follow-up, and data collection	2
Participants	6	(*a*) Give the eligibility criteria, and the sources and methods of selection of participants. Describe methods of follow-up	2, Fig S1
		(*b*) For matched studies, give matching criteria and number of exposed and unexposed	Tbl 1, Tbl S5
Variables	7	Clearly define all outcomes, exposures, predictors, potential confounders, and effect modifiers. Give diagnostic criteria, if applicable	2
Data sources/ measurement	8*	For each variable of interest, give sources of data and details of methods of assessment (measurement). Describe comparability of assessment methods if there is more than one group	2
Bias	9	Describe any efforts to address potential sources of bias	12
Study size	10	Explain how the study size was arrived at	2
Quantitative variables	11	Explain how quantitative variables were handled in the analyses. If applicable, describe which groupings were chosen and why	3
Statistical methods	12	(*a*) Describe all statistical methods, including those used to control for confounding	3
		(*b*) Describe any methods used to examine subgroups and interactions	N/A
		(*c*) Explain how missing data were addressed	N/A
		(*d*) If applicable, explain how loss to follow-up was addressed	N/A
		(*e*) Describe any sensitivity analyses	N/A

medRxiv preprint doi: https://doi.org/10.1101/2023.12.18.23300042; this version posted December 18, 2023. The copyright holder for this preprint (which was not certified by peer review) is the author/funder, who has granted medRxiv a license to display the preprint in perpetuity.

It is made available under a CC-BY 4.0 International license .

**Results**			
Participants	13*	(a) Report numbers of individuals at each stage of study—eg numbers potentially eligible, examined for eligibility, confirmed eligible, included in the study, completing follow-up, and analysed	Fig S1
		(b) Give reasons for non-participation at each stage	Fig S1
		(c) Consider use of a flow diagram	Fig S1
Descriptive data	14*	(a) Give characteristics of study participants (eg demographic, clinical, social) and information on exposures and potential confounders	Tbl 1
		(b) Indicate number of participants with missing data for each variable of interest	Tbl S2, S3, S5
		(c) Summarise follow-up time (eg, average and total amount)	Tbl 1
Outcome data	15*	Report numbers of outcome events or summary measures over time	1, 2, Tbl 1

Main results	16	(*a*) Give unadjusted estimates and, if applicable, confounder-adjusted estimates and their precision (eg, 95% confidence interval). Make clear which confounders were adjusted for and why they were included	Tbl S1, Fig 1
		(*b*) Report category boundaries when continuous variables were categorized	N/A
		(*c*) If relevant, consider translating estimates of relative risk into absolute risk for a meaningful time period	N/A
Other analyses	17	Report other analyses done—eg analyses of subgroups and interactions, and sensitivity analyses	N/A
**Discussion**			
Key results	18	Summarise key results with reference to study objectives	3-4
Limitations	19	Discuss limitations of the study, taking into account sources of potential bias or imprecision. Discuss both direction and magnitude of any potential bias	4
Interpretation	20	Give a cautious overall interpretation of results considering objectives, limitations, multiplicity of analyses, results from similar studies, and other relevant evidence	4
Generalisability	21	Discuss the generalisability (external validity) of the study results	4
**Other information**			
Funding	22	Give the source of funding and the role of the funders for the present study and, if applicable, for the original study on which the present article is based	5
